# Identifying Gaps and Launching Resident Wellness Initiatives: The 2017 Resident Wellness Consensus Summit

**DOI:** 10.5811/westjem.2017.11.36240

**Published:** 2018-02-19

**Authors:** Fareen Zaver, Nicole Battaglioli, William Denq, Anne Messman, Arlene Chung, Michelle Lin, Emberlynn L. Liu

**Affiliations:** * University of Calgary, Department of Emergency Medicine, Alberta, Canada; † Mayo Clinic, Department of Emergency Medicine, Rochester, Minnesota; ‡ George Washington University, Department of Emergency Medicine, Washington, District of Columbia; § Sinai-Grace Hospital, Detroit Medical Center, Department of Emergency Medicine, Detroit, Michigan; ¶ Mount Sinai Hospital, Department of Emergency Medicine, New York, New York; || University of California San Francisco, Department of Emergency Medicine, San Francisco, California; ** University of Texas Southwestern, Department of Emergency Medicine, Dallas, Texas

## Abstract

**Introduction:**

Burnout, depression, and suicidality among residents of all specialties have become a critical focus for the medical education community, especially among learners in graduate medical education. In 2017 the Accreditation Council for Graduate Medical Education (ACGME) updated the Common Program Requirements to focus more on resident wellbeing. To address this issue, one working group from the 2017 Resident Wellness Consensus Summit (RWCS) focused on wellness program innovations and initiatives in emergency medicine (EM) residency programs.

**Methods:**

Over a seven-month period leading up to the RWCS event, the Programmatic Initiatives workgroup convened virtually in the Wellness Think Tank, an online, resident community consisting of 142 residents from 100 EM residencies in North America. A 15-person subgroup (13 residents, two faculty facilitators) met at the RWCS to develop a public, central repository of initiatives for programs, as well as tools to assist programs in identifying gaps in their overarching wellness programs.

**Results:**

An online submission form and central database of wellness initiatives were created and accessible to the public. Wellness Think Tank members collected an initial 36 submissions for the database by the time of the RWCS event. Based on general workplace, needs-assessment tools on employee wellbeing and Kern’s model for curriculum development, a resident-based needs-assessment survey and an implementation worksheet were created to assist residency programs in wellness program development.

**Conclusion:**

The Programmatic Initiatives workgroup from the resident-driven RWCS event created tools to assist EM residency programs in identifying existing initiatives and gaps in their wellness programs to meet the ACGME’s expanded focus on resident wellbeing.

## INTRODUCTION

Burnout, depression, and suicidality among residents of all specialties have become a critical focus of attention for the medical education community. Prevalence studies have revealed rates of burnout among residents to be as high as 76% as measured by the Maslach Burnout Inventory (MBI).^1^ In response to these findings, the Accreditation Council for Graduate Medical Education (ACGME) approved major changes to the Common Program Requirements in 2017. These changes establish a mandate to educate residents and faculty members in the identification of burnout, depression, and substance abuse and for implementing programs that encourage optimal resident and faculty wellbeing.^2^ There are however, no roadmaps or guidelines for residency programs to create such wellness programs to adequately address this mandate.

Many residency programs have already implemented wellness training and initiatives for their residents. Unfortunately, evidence supporting the efficacy of these interventions is sparse and often limited to single institutions and small sample sizes.^3^,^4^ Furthermore, there is no established method of sharing preliminary experiences and lessons learned from these interventions with other residency programs also seeking to improve their wellness curricula.

The 2017 Resident Wellness Consensus Summit (RWCS)^5^ convened as a pre-day to a national emergency medicine (EM) conference, Essentials of Emergency Medicine, to address many aspects of resident wellness and burnout. One of the working groups, Programmatic Initiatives, focused specifically on starting an online, crowdsourced, central repository of wellness initiatives in EM residency programs. Additionally, the working group aimed to develop a resident-based needs assessment and implementation instrument to assist programs launch their own wellness programs.

## METHODS

In October 2016 a volunteer group of 142 EM residents from 100 training programs across North America formed the Wellness Think Tank, a virtual community of practice focusing specifically on resident wellness. All EM residency programs in North America were invited to enroll up to two EM residents as representatives in the Think Tank. Members of this online community, hosted by a medical education organization Academic Life in Emergency Medicine (ALiEM), communicated with each other using the online platform #Slack. On this shared workspace platform, members discussed the strengths and weaknesses of wellness programs at their respective training sites. During these discussions, residents noted duplicated efforts at different programs and a siloed approach to wellness initiatives, which they attributed primarily to a lack of shared knowledge among residency programs.

All participating residents of the Wellness Think Tank as well as the broader EM resident population in the United States were invited to the in-person RWCS event on May 15, 2017 (Las Vegas, NV).^5^ In preparation for the event, a Programmatic Initiatives working group was created within the Wellness Think Tank to develop an initial, centralized, crowdsourced database of existing wellness strategies in EM residency programs. Members of the Wellness Think Tank and the Chief Resident Incubator, another virtual community of practice hosted by ALiEM, were asked to contribute submissions about their local wellness strategies, specifically describing the resources required, whether the initiative or event was child-friendly, and practical implementation tips. A total of 22 resident members from the Wellness Think Tank, and 22 additional EM residents attended the live RWCS event. Of the 44 residents, 13 residents (as well as two faculty facilitators selected by the ALiEM leadership team for their facilitation expertise) served as the final Programmatic Initiatives working group. At the RWCS event, the working group reviewed the residency program initiatives in the database and developed two tools for residency programs – a resident-based, needs-assessment tool to identify gaps in wellness programming and a systematic worksheet to help programs implement new wellness initiatives. Following the RWCS event, the database and tools were further refined based on feedback, ideas, and comments from the Wellness Think Tank resulting in the final versions presented here.

## RESULTS

The Programmatic Initiatives working group identified an initial 36 unique residency wellness initiatives, collected from the Wellness Think Tank and Chief Resident Incubator communities. These initiatives are listed in a centralized, searchable, online database open to the public along with a contributor form for future submissions at https://www.aliem.com/wellness-think-tank/wellness-initiatives-database/.

The working group also developed two tools. The first tool is a Resident-Based Needs Assessment Survey on residency wellness programming (Appendix A). This survey should be administered to individual residents to inform programwide strategic planning on wellness activities. This tool was created based on a framework modeled after existing needs-assessment tools on employee wellness in the general workplace.^6^,^7^ The resident needs assessment systematically evaluates the current wellness initiatives in a program, existing wellness interests of the residents, the perception of the culture of wellness, and leadership support for wellness activities. Open-ended questions were included throughout the survey to capture suggestions or further input from residents to encourage creative responses and novel ideas. Although some programs may have a wellness program already in place, the tool can still be used on a yearly basis to help programs adjust based on evolving resident needs.

The second tool (Worksheet on Implementing New Wellness Initiatives) is a systematic worksheet to help residency programs implement new wellness initiatives (Appendix B). Using the principles from Kern’s six-step model for curriculum development,^8^ the worksheet is divided into two parts. Part I explores existing resources and previous experiences with wellness initiatives in one’s program and at the broader institutional level in a targeted, needs-assessment approach. Part II then focuses on building one, new wellness initiative or strategy. This guides readers to familiarize themselves with stakeholders and potential obstacles to implementation by addressing educational strategies, resource identification, implementation barriers, and outcome measures. Unanswered questions should be addressed before investing time and resources to the initiatives. A sample completed worksheet on developing a resident mentorship program is also included as a guide.

## DISCUSSION

Although physicians of all specialties are at increased risk of depression and suicide, emergency physicians are among those at greatest risk.^9^–^13^ Furthermore, burnout rates are high for medical students, residents, and early-career physicians across specialties.^14^ To address this, the resident-driven 2017 RWCS event and 2016–17 Wellness Think Tank community focused on developing a consensus on various wellness issues and problems deemed high priority by EM residents. Through online discussions leading up to the RWCS event, residents realized multiple instances of duplicated wellness initiatives at different programs with little to no sharing of their experiences. Thus, the Programmatic Initiatives working group first focused on identifying and publicly sharing existing wellness activities in EM residency programs. The group also assisted programs launching new wellness initiatives and strategies. Our hope is that these collective resources serve as a framework for EM residency programs seeking guidance in meeting the 2017 ACGME Common Program Requirement mandate to build a robust infrastructure and educational strategy to address resident and faculty wellbeing.^2^

## CONCLUSION

The Programmatic Initiatives working group for an EM resident-driven consensus conference tackled the specific issues of sharing existing wellness initiatives and creating instruments to help residency programs thoughtfully plan and implement new wellness initiatives.

## Supplementary Information




