# Supplementary material for: Identifying Gaps and Launching Resident Wellness Initiatives: The 2017 Resident Wellness Consensus Summit
Source: West J Emerg Med. 2018 Feb 19;19(2):342–5. doi: 10.5811/westjem.2017.11.36240 (PMC5851509; doi:10.5811/westjem.2017.11.36240)
Supplement: Supplementary file 1 [file wjem-19-342-s001.pdf]

# Needs Assessment Survey on Wellness for a Residency Program

## 2017 Resident Wellness Consensus Summit

By Zaver F, Battaglioli N, Denq W, Messman A, Chung A, Liu EL
Appendix A to publication: … *[insert WestJEM citation]*

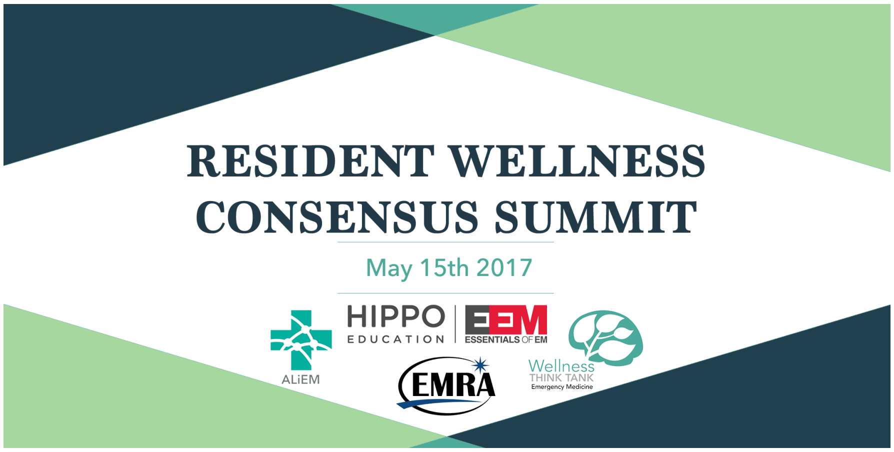

# Instructions

This needs assessment survey should be administered to individual residents anonymously. The aggregate results should inform the residency program in implementing wellness activities and improving the culture of wellness.

# Needs Assessment Survey to Improve Residency Wellness

Thank you for helping the residency program to improve the state and culture of wellness in this anonymous needs assessment survey. Please be as specific and honest as possible in your responses.

1. **Overall, how would you rate your level of burnout?** Please select one.
   - ☐ I have no symptoms of burnout.
   - ☐ I am occasionally under stress. I don't always have as much energy as I once did, but I do not feel burned out.
   - ☐ I am definitely burning out, and have one or more symptoms of burnout such as physical or emotional exhaustion.
   - ☐ The symptoms of burnout that I am experience will not go away. I think about frustration at work a lot.
   - ☐ I feel completed burned out and often wonder if I can go on. I am at the point where I may need some changes or may need to seek some sort of help.
   - ☐ Other: ______________________________________________________________

2. **Do you feel that there is a culture of wellness in our program?** Select one.
   - ☐ Yes
   - ☐ No
   - ☐ Not sure

3. **How satisfied are you with the current wellness initiatives in our program?** Select one.
   - ☐ 1 (not very satisfied)
   - ☐ 2
   - ☐ 3
   - ☐ 4
   - ☐ 5 (very satisfied)

Wellness
THINK TANK
Emergency Medicine

4. **How satisfied are you with the following components and resources in our residency program?** Select one box per row.

| | Not satisfied | Somewhat satisfied | Satisfied | Very satisfied |
|---|---|---|---|---|
| Work hours | ☐ | ☐ | ☐ | ☐ |
| Professional development/ Leadership skills | ☐ | ☐ | ☐ | ☐ |
| Food and nutrition | ☐ | ☐ | ☐ | ☐ |
| Exercise and sports | ☐ | ☐ | ☐ | ☐ |
| Debriefing and reflection | ☐ | ☐ | ☐ | ☐ |
| Counseling | ☐ | ☐ | ☐ | ☐ |
| Family support and events | ☐ | ☐ | ☐ | ☐ |
| Social activities | ☐ | ☐ | ☐ | ☐ |
| Mentorship | ☐ | ☐ | ☐ | ☐ |
| Financial advising | ☐ | ☐ | ☐ | ☐ |

5. **What types of wellness initiatives would you most interested in participating in/helping to organize?** Select one box per row.

| | Not interested | Somewhat interested | Interested | Very interested |
|---|---|---|---|---|
| Personal improvement | ☐ | ☐ | ☐ | ☐ |
| Professional development | ☐ | ☐ | ☐ | ☐ |
| Food and nutrition | ☐ | ☐ | ☐ | ☐ |
| Exercise and sports | ☐ | ☐ | ☐ | ☐ |
| Debriefing and reflection | ☐ | ☐ | ☐ | ☐ |
| Counseling | ☐ | ☐ | ☐ | ☐ |
| Family support and events | ☐ | ☐ | ☐ | ☐ |

6. **What would be one thing to change AT the workplace to make things less stressful?**

Wellness
THINK TANK
Emergency Medicine

7. **What would be one thing to change OUTSIDE the workplace, to further improve camaraderie or tackle common non-medical challenges faced by residents?**

8. **How would you like to hear about ongoing and upcoming wellness initiatives?** Check all that apply.

- ☐ Announcement at residency conference
- ☐ Email
- ☐ Messaging app (e.g. GroupMe, Slack, Whatsapp)
- ☐ Newsletter
- ☐ Social media
- ☐ Other: ____________________________________

9. **Please provide any additional feedback for the residency leadership and/or wellness committee.**
