# Supplementary material for: Identifying Gaps and Launching Resident Wellness Initiatives: The 2017 Resident Wellness Consensus Summit
Source: West J Emerg Med. 2018 Feb 19;19(2):342–5. doi: 10.5811/westjem.2017.11.36240 (PMC5851509; doi:10.5811/westjem.2017.11.36240)
Supplement: Supplementary file 2 [file wjem-19-342-s002.pdf]

Worksheet on Implementing New Wellness Initiatives in a Residency Program

2017 Resident Wellness Consensus Summit

By Zaver F, Battaglioli N, Denq W, Messman A, Chung A, Liu EL

Appendix B to publication: … *[insert WestJEM citation]*

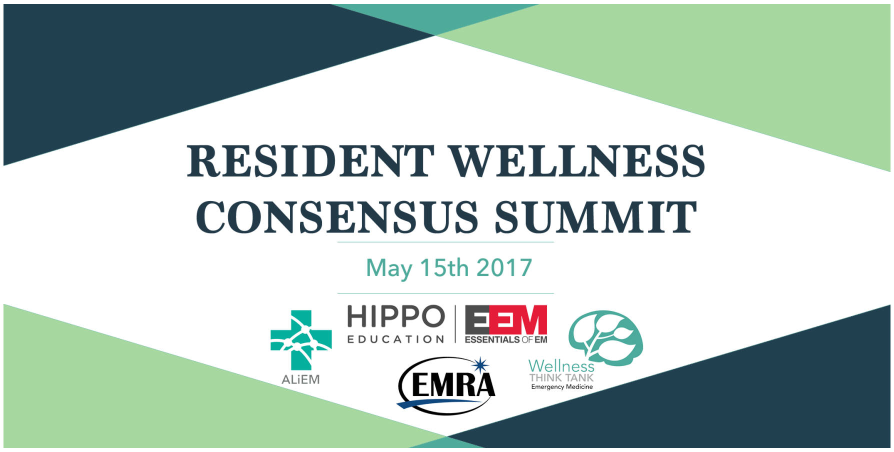

Instructions

This worksheet systematically evaluates existing resources and potential barriers within one's program or department in launching wellness initiatives. The person or group in charge of implementing such initiatives at the program level should complete this form.

Part I is a general form assessing the overall state of wellness initiatives in one's program, department, and/or institution. **Part II** provides an inquiry-based, step-wise approach on how to implement a specific new wellness initiative. Included is an example of a completed form on specifically improving resident mentorship.

Part I: Program Overview

1. How is wellness currently being addressed in your program and/or department?

2. What has successfully been implemented in your program and/or department?

3. What wellness initiatives are already exist in other specialties at your institution?

4. Are you able to share resources with other programs at your institution? If so, please explain.

Wellness THINK TANK Emergency Medicine

Part II: Implementing a Specific Wellness Initiative

1. **Identify one specific issue in resident wellness that needs improvement.**

2. **Who are the stakeholders?**

NOTE: Be aware that one's hospital, medical center, medical school, departments, and residency programs may function separately or synergistically.

Stakeholder	How are they impacted by launching the wellness initiative?

3. **Who are the skeptics or obstructionists? Why do they take such a position?**

Wellness THINK TANK Emergency Medicine

4. **What are strategies to approach these skeptics or obstructionists?**

5. **Identify the expert individual(s) available in your residency program, department, or institution to address the wellness initiative issue.**

6. **Independent of resources, funding, and time, what would be your ideal approach to address the wellness initiative issue?**

7. **What are expected barriers to implementing the wellness initiative?**

Wellness
THINK TANK
Emergency Medicine

8. **What are potential solutions to address these barriers?**

9. **Describe the financial and administrative support available for the wellness initiative.**

10. **What is the measure of success, and how will it be measured? What is the feedback mechanism in evaluating these measures?**

11. **If there currently is no funding for the proposed wellness initiative, how do you plan on addressing any funding needs?** *(For instance, is the funding to come from a philanthropist, institution, hospital, department, residency program, or individual residents?)*

Wellness THINK TANK Emergency Medicine

Example:
Implementing a Wellness Initiative on Resident Mentorship

1. **Identify one specific issue in resident wellness that needs improvement.**

Residents have requested opportunities for more formal mentorship.

2. **Who are the stakeholders?**

**Stakeholder**	**How are they impacted by a resident mentorship program?**
Resident	Improved guidance in advancing successfully through residency training, broadened network of colleagues and collaborators, and richer insights into the pros/cons of potential career paths.
Faculty	Opportunity to provide mentorship based on career experiences and, in doing so, further develop leadership skills; mentorship is often a required component for faculty promotions in academic institutions
Residency program	A mentorship program may be an attractive feature for the program to advertise to prospective medical student applicants. More successful, well-informed, and happy residents can help maintain year-round positive program morale and less burnout.
Department	Attractive element of faculty development
Patients	Improved physician-patient relationships due to reduced levels of resident burnout

3. **Who are the skeptics or obstructionists? Why do they take such a position?**

Residents may not see the value of a formal mentorship program. They may claim that they do not have the time, interest, or trust in the imbalanced relationship in the power hierarchy (e.g. Sharing something personal may have a negative impact on one's formal evaluation). **Faculty** may not feel mentorship is helpful or necessary for residents. They may feel that they do not have time for an additional academic responsibility.

4. **What are strategies to approach these skeptics or obstructionists?**

Resident - Match residents with mentors who share a common professional interest and are not directly involved with their evaluations. Provide dedicated non-clinical time for mentorship meetings.

Faculty – Highlight the benefits of mentoring a resident with shared professional interests (e.g. a potential future collaborator, a source of fresh new insights into old ideas, paying it forward for received mentorship). Outline the requirements for academic promotions involving active mentorship. Provide dedicated non-clinical time for mentorship meetings.

5. **Identify the expert individual(s) available in your residency program, department, or institution to address the wellness initiative issue.**

Dr. XXX is the Director of Faculty Development in the department and has voiced interest in helping to establish a more formal, resident-faculty paired mentorship program based on their mutual academic, research, education, or other career interests. Furthermore, she has a broad network of colleagues outside of the department and institution, who may able to also serve as additional faculty mentors for the residents, based on their professional interests and needs.

6. **Independent of resources, funding, and time, what would be your ideal approach to address the wellness initiative issue?**

A formal mentorship program would involve a resident-faculty dyad model. This pair would meet periodically in a non-judgmental fashion with no impact on resident summative evaluations. A broad range of topics would cover not only career counseling but also any individual resident wellness, resiliency, or burnout issues, which the faculty member would tactfully help to normalize, provide thoughtful reflection, and contribute stories of similar personal experiences.

These formal biannual meetings may occur in person or by videochat in a casual setting. A pre-designed list of icebreaker topics would be made available to the mentor and mentee. There would also be administrative support to remind the mentor to periodically contact the resident via email or text messages to check in.

7. **What are expected barriers to implementing the wellness initiative?**

Availability of the resident and faculty member
Personality fit between the faculty mentor and the resident
Unclear discussion topics
Uncertain how to establish psychological safety and trust in this mentorship pairing
Cost

8. **What are potential solutions to address these barriers?**

Protected time: At the beginning of the academic year, schedule the meeting dates/times for the entire year to minimize any potential scheduling conflicts. Consider scheduling the meetings during the residency conference block because residents are already released from clinical duties.
Personality fit: Obtain feedback from the mentor and mentee on whether the pairing is a good fit every year. Encourage pairings to change, if needed.
Discussion topics: Provide mentors and mentees with a list of potential discussion points
Trust: Conduct the sessions in informal locations (e.g. coffee house) and encourage the faculty member to share personal stories to augment/supplement the discussions. Provide the faculty members and the residents with literature on optimizing mentorship experiences.
Cost: Allow faculty to use their CME funds to pay for coffee and snacks for the residents at their meetup.

9. **Describe the financial and administrative support available for the wellness initiative.**

The residency coordinator has agreed to help identify the biannual meeting dates and times. He will also be sending quarterly reminders to the faculty members to text or email the resident to check in.

10. **What is the measure of success, and how will it be measured? What is the feedback mechanism in evaluating these measures?**

Success will be measured based on an end-of-year survey of the mentors and mentees, assessing personality fit, goal-setting process, and personal/professional growth. Additionally they will be asked to highlight effective practices and discussion questions that can contribute to a "how to be an effective mentor-mentee" handout for the entire department.

11. **If there currently is no funding for the proposed wellness initiative, how do you plan on addressing any funding needs?**

If the year-end evaluations (see #10) are consistently highly rated, the residency leadership could be approached to purchase some coffee house gift cards for each mentor to use for each meeting. Alternatively, the residents could award the best mentors, whereby the prize would be a coffee house gift card that could be used for future mentorship meetings.
